# Flow-following sensor devices: A tool for bridging data and model predictions in large-scale fermentations

**DOI:** 10.1016/j.csbj.2020.10.004

**Published:** 2020-10-15

**Authors:** Jonas Bisgaard, Monica Muldbak, Sjef Cornelissen, Tannaz Tajsoleiman, Jakob K. Huusom, Tue Rasmussen, Krist V. Gernaey

**Affiliations:** a Freesense ApS, Copenhagen, Denmark; b Process and Systems Engineering Center (PROSYS), Department of Chemical and Biochemical Engineering, Technical University of Denmark, Building 228A, 2800 Kgs. Lyngby, Denmark; c Novozymes A/S, Bagsværd, Denmark

**Keywords:** Sensor device, Flow-follower, Modelling, Computational fluid dynamics, Model validation, Mixing, Gradients, Large-scale bioreactor, Industrial biotechnology

## Abstract

Production-scale fermentation processes in industrial biotechnology experience gradients in process variables, such as dissolved gases, pH and substrate concentrations, which can potentially affect the production organism and therefore the yield and profitability of the processes. However, the extent of the heterogeneity is unclear, as it is currently a challenge at large scale to obtain representative measurements from different zones of the reactor volume. Computational fluid dynamics (CFD) models have proven to be a valuable tool for better understanding the environment inside bioreactors. Without detailed measurements to support the CFD predictions, the validity of CFD models is debatable. A promising technology to obtain such measurements from different zones in the bioreactors are flow-following sensor devices, whose development has recently benefitted from advancements in microelectronics and sensor technology. This paper presents the state of the art within flow-following sensor device technology and addresses how the technology can be used in large-scale bioreactors to improve the understanding of the process itself and to test the validity of detailed computational models of the bioreactors in the future.

## Graphical abstract

**Figure:**
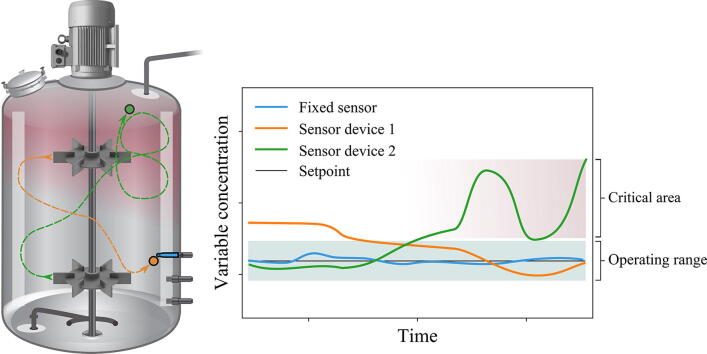

## 1 Introduction

Fermentations in bioreactors form the core technology of a huge and rapidly growing market, which is supplying products such as amino acids, organic acids, polymers, enzymes, vitamins, antibiotics, biopharmaceuticals, and starter cultures for food production [1]. Most of the production is carried out in submerged fermentations, which are broths consisting of growth media and a given production microorganism of bacterial or fungal origin or a mammalian cell culture. The broth is contained in large stainless-steel bioreactors, ranging from 20 m^3^ to 2000 m^3^ [2], whose purpose it is to provide an optimal and axenic environment for the cells to grow and produce. Together with the appropriate mixture of nutrients and availability of molecular oxygen (for aerobic processes), specific values for physical parameters such as pH and temperature are crucial for an optimal environment. Therefore, the bioreactor is purposefully designed to ensure mixing of the broth, to ensure efficient gas-to-liquid mass transfer for oxygen, and to guarantee efficient heat removal [3]. During aerobic growth, the temperature rises because metabolic heat is released. The growth also leads to a rise in oxygen consumption by the cells which increases the overall oxygen demand in the bioreactor. In addition, metabolites altering the pH of the broth may be produced. All these changes must be counteracted by a proper control system. Additionally, most industrial fermentation processes are run in fed-batch mode [4], which means that a substrate solution is fed to the broth at a controlled rate, such that one substrate component is growth rate limiting. The advantage of this approach is that the reaction rate can be controlled using the dosing rate to avoid engineering limitations with respect to cooling and oxygen transfer and to avoid by-product formation [4]. Measurements from sensors in the bioreactor such as temperature, pH and DO sensors and offline samples from fixed sample ports, are typically taken at only one point in the bioreactor [5]. This is of concern because it is technically infeasible to obtain ideal mixing in large-scale bioreactors. Hence, due to an interplay between the rates of mixing, mass transfer and microbial reactions, such as substrate consumption and growth rate, heterogeneities exist within the bioreactor. Measuring or taking samples from a single specific point in the reactor are therefore strategies that are likely to be non-representative for the entire reactor volume. The heterogeneities may affect process performance because the cells experience fluctuations between favorable and unfavorable environments during their trajectories in the bioreactor. These environmental fluctuations can lead to a decreased product yield, reduced volumetric productivity and/or decreased product quality compared to products obtained from operation of smaller-scale bioreactors, in which the process has been developed and piloted [6], [7].

The presence of gradients of important process variables, such as substrate concentration and DO concentration, have been demonstrated by measuring or extracting samples from multiple locations in large-scale bioreactors [8], [9], [10] using custom built sensor fixtures or sampling systems [11]. Nowadays, however, most knowledge of heterogeneity in large-scale bioreactors is obtained through computational methods, such as computational fluid dynamics (CFD) models together with kinetic models or advanced cell models [12]. These models offer a more detailed understanding of the relevant phenomena in the bioreactor environment than the local measurements [13]. However, high-quality experimental data is fundamental to make rational model assumptions and to validate these models, but only a handful of such data-sets from large-scale bioreactors are available in the scientific literature [14]. This is a concern as CFD studies of large-scale reactors provide little [10], [15], [16] or in some cases no experimental validation [17], [18], [19]. Since the pioneering work by Lapin et al [20], the Euler-Lagrange approach has been used to simulate environmental fluctuations from the perspective of a microorganism, or more precisely a group of microorganisms. By simulating microorganisms as particles, it is possible to extract time series of a variable of interest, such as substrate concentration or DO concentration, associated with the trajectories of the particles. The extracted time series, which can be interpreted as the microorganisms perception of the variation of given variables in time, are referred to as lifelines [20], and can be statistically analyzed to provide a comprehensive insight of the environmental fluctuations experienced by the microorganisms [15].

**Fig. 1:**
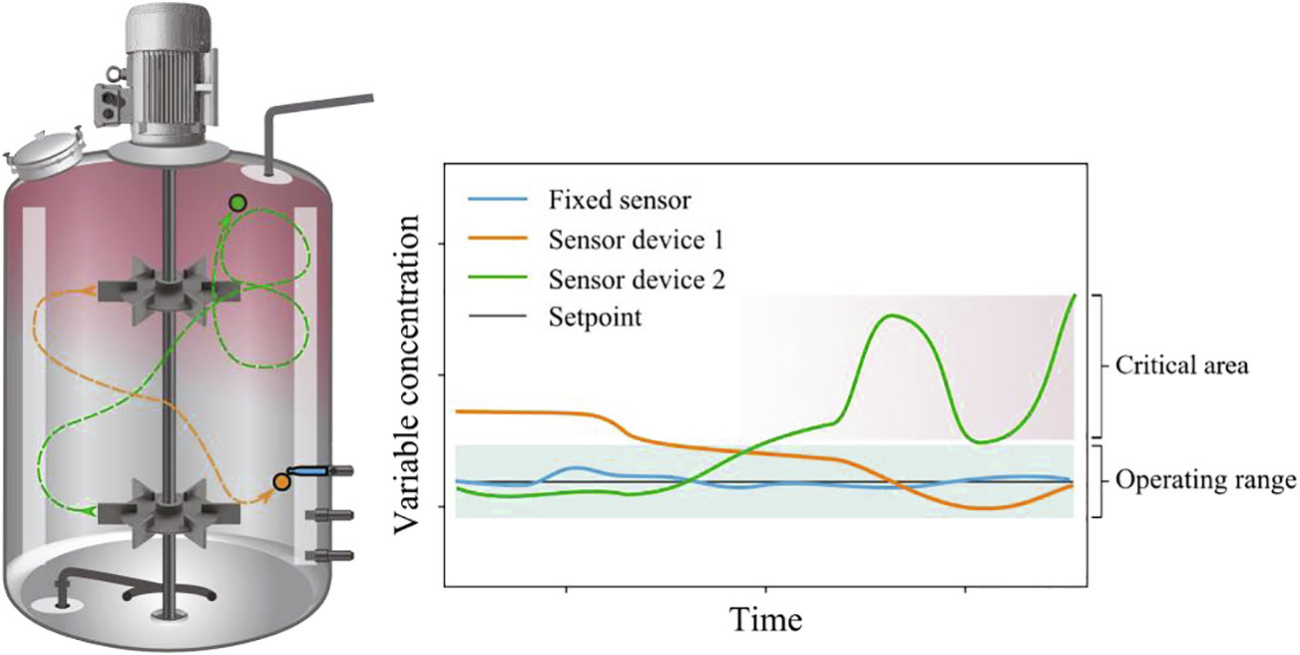
Illustration of the use of sensor devices in a large-scale stirred tank bioreactor. The sensor devices will be carried around with the liquid flow and measure relevant variables that potentially represent what microorganisms would experience when travelling throughout the volume. The measured values may deviate from the normal operating range in certain zones of the reactor volume.

The same trend of studying fluctuating environments through particles is present within measurement technology, where a new generation of Lagrangian measurement techniques, the autonomous, instrumented and flow-following sensor devices, are emerging. Generally speaking, Lagrangian measurement techniques make use of particles, which are embedded into the flow itself and carried along with the agitation or convection induced fluid motion [21], [22]. Compared to established Lagrangian measurement techniques, such as positron emission particle tracking (PEPT) and computer-aided radioactive particle tracking (CARPT), the sensor devices are particularly suitable for bioreactors because of their ability to autonomously measure and store/transmit data which significantly simplifies the experimental procedures. Moreover, the sensor devices are equipped with one or more sensors to measure variables, such as temperature, pH, DO, etc. In addition, sensors that provide information about the position of the sensor device may be integrated. A simple example of position tracking is a pressure sensor that provides information about the immersion depth [23], thus allowing for analysis of the bioreactor environment in both space and time. As an example of the concept, Fig. 1 illustrates a heterogeneous steady state bioreactor, with a gradient of some arbitrary variable representing a compound that is continuously added at the top and consumed within the reactor.

In such a system, the fixed sensor measures a steady value around the setpoint, while the sensor devices move to zones where the variable concentration could be critically high or critically low. This means that the variable concentration deviates from the normal operating or quality range in certain zones of the reactor volume, which could represent a major quality concern. Sensor devices may therefore serve as a process analytical technology (PAT), to monitor critical process parameters (CPPs) during pharmaceutical manufacturing.

The aim of this paper is to present the state-of-the-art within sensor device technology, and to discuss how this emerging technology can be applied in large-scale bioreactors to gather detailed spatio-temporal information and how this information can be merged with computational models that have been developed for large-scale bioreactors.

The paper is organized as follows: Section two extends the introduction with some considerations about flow following capabilities, which are important when sensor devices are used to examine flows or when the measurements are interpreted as what the microorganisms experience. Section three presents the development of Langrangian technologies leading to the state-of-the-art flow-following sensor devices, while addressing some advantages and limitations of the different technologies. Section four contains a discussion on the application of flow-following sensor devices in large-scale bioreactors and how they synergize with current state of the art CFD models. Finally, a summary of this review paper is provided combined with an outlook towards future development of the technology.

## 2 Flow following capabilities

Recent CFD studies of large-scale bioreactors have shown a considerable interest towards understanding the fluctuations in process variables that the microorganisms may experience in the reactors [15], [17], [18], [19]. It is therefore also obvious to interpret measurements from Lagrangian techniques as if they were microorganisms moving around with the liquid streamlines in the bioreactor. Lagrangian techniques have also proven useful in studies of flow and mixing [24], [25], [26], [27], [28], especially the Lagrangian techniques which are appropriate for the use in large-scale bioreactors [26]. No experimental approaches are readily available to study the flows in large-scale bioreactors in detail [29] and the knowledge about mixing is often limited to the mixing time [22]. Exactly how the information obtained by Lagrangian techniques can be used to study flow and mixing in bioreactors may vary with the individual technology. These methods will be covered when introducing the Lagrangian technologies in the following section. However, for any of these applications to be justified, the particles must exhibit flow following behavior. Lagrangian techniques having this trait are also known as flow-followers.

The Lagrangian particles behave more like the liquid when they are neutrally buoyant, i.e. when the density of the particles is equal to the surrounding liquid. However, the condition of neutral buoyancy poses a problem in aerated bioreactors, where the apparent density of the gas–liquid dispersion is reduced when air is introduced. The apparent density of the gas–liquid dispersion is calculated as *ρ_app_* = (1 − *ε*)*ρ_l_* − *ερ_g_*, where *ρ_l_* is the liquid density, *ρ_g_* the gas density, and ε the volumetric gas-holdup [30]. The term *ερ_g_* is often negligible as the gas density is only a fraction of the liquid density at fermentation conditions. In such a system, Middleton [31] showed that flow-following particles which are large compared to the mean bubble size, experience the mean density of the gas–liquid dispersion, whereas flow following particles that are smaller than the mean bubble size, experience the liquid density. Therefore, large particles (i.e. larger than the mean bubbles size) that are neutrally buoyant in a liquid, will be settling with a given velocity when aeration is introduced in the liquid.

Besides experiencing minimal effect from buoyancy or settling forces on the velocity, the particles must also be able to respond to the changes in the velocity field. If the response of the particle is too slow, the particle will detach from the liquid streamlines and will therefore represent the hydrodynamics inaccurately. Moreover, if lifeline studies are the motivation, the environment that the particle experiences will only have a weak relation to the environment that a microorganism would experience. The trait of being able to respond to changes in the velocity field can be quantified by the Stokes Number: *St = τ_p_/τ_f_*, where *τ_p_* represents the momentum response time of a particle and *τ_f_* is some time characteristic of the flow field [32]. *St* ≪ 1 means that the response time of the particles to changes in the velocity field is much lower than the characteristic time associated with the flow field; therefore, the particle behaves like the fluid, which is exactly what is desired for flow-following particles. On the other hand, if *St* ≫ 1, then the particle will have no time to respond to the fluid velocity changes and will likely detach from the fluid streamlines. From a practical point of view, it can be stated that the condition St < 0.1, returns an acceptable flow tracing accuracy with errors below 1% [32]. A generalized form of the equation for momentum response time is shown in Eq. (1) [33].

(1) $$\tau_{p}=\frac{4}{3}\cdot \frac{\rho_{p}\cdot d_{p}^{2}}{\mu_{f}\cdot Re_{p}\cdot C_{D}\left(Re_{p}\right)},$$

with the Reynolds number for particles *Re_p_* defined in Eq. (2).

(2) $$Re_{p}=\frac{\rho_{f}\cdot d_{p}\cdot \left|u-v\right|}{\mu_{f}}.$$

Eqs. (1), (2) can be combined to obtain Eq. (3).

(3) $$\tau_{p}=\frac{4}{3}\cdot \frac{\rho_{p}}{\rho_{f}}\cdot \frac{d_{p}}{\left|u-v\right|C_{D}\left(Re_{p}\right)},$$

where *ρ_p_* (kg/m^3^) and *ρ_f_* (kg/m^3^) are the particle density and fluid density, respectively, *d_p_* (m) is the particle diameter, *C_D_* (–) the drag coefficient, *v* (m/s) the particle velocity and *u* (m/s) the fluid velocity [33].

From this Eq. (3) it is apparent that a reduction of the particle diameter leads to a reduction in the momentum response time and therefore a reduction in Stokes number. Hence, this is leading to the intuitive conclusion that smaller particles have better flow following capabilities. This becomes important with respect to sensor devices, which due to the incorporation of batteries, electronics and sensor technology, face limitations with respect to the diameter. In this case, a lower tracing accuracy may be considered acceptable but should be taken into consideration when interpreting the results. In contrast to a smaller diameter, a higher drag coefficient leads to better flow following capabilities, as this lowers the momentum response time and the Stokes number. The drag coefficient is associated with the contribution from particle shape and orientation on the hydrodynamic drag. A higher drag coefficient implies a higher drag force acting on the particle. The drag coefficient is not a constant, but rather a function of Reynolds number. In fact, the drag coefficient decreases with increasing Reynolds number, e.g. from increasing the particle diameter [33], [34]. Nonetheless, the overall contribution from an increased particle diameter still results in a higher momentum response time. Increasing viscosity, on the other hand, results in a higher drag coefficient and an overall reduction of the momentum response time and the Stokes number. The contribution from drag on the flow-following capabilities makes sense as the term “flow-following” entails that the particle is getting dragged along with the liquid which has forces acting in the direction of the flow.

## 3 Evolution of Lagrangian technologies for bioreactors

**Fig. 2:**
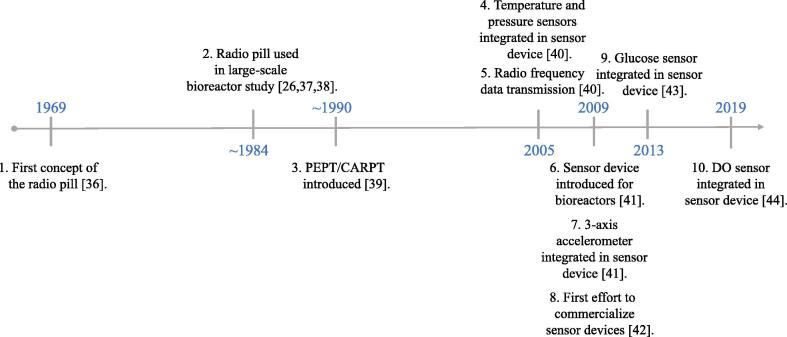
Timeline highlighting important developments in Lagrangian technologies for bioreactors. (See above-mentioned reference for further information.)

Lagrangian technologies, and more specifically sensor devices, have developed rapidly in the last decades, which is apparent from the timeline in Fig. 2; the timeline highlights important developments within the area. Advances in nanofabrication have significantly reduced the size of integrated circuits, enabling the miniaturization of electronics, while advances within micromachining have led to the production of microelectromechanical systems (MEMS) and microsensors [35]. These technologies constitute key components in the state-of-the-art sensor devices, with some examples of MEMS being pressure sensors and accelerometers. Examples of the microsensors include certain types of pH and dissolved oxygen sensors.

The application of Lagrangian sensor technologies in industrial biotechnology dates back to the late sixties where J. Bryant developed a radio pill for his PhD thesis in 1969 [36]. The radio pill was a plastic encapsulated and battery powered radio transmitter, which could be detected when the radio signal was picked up by a proximate antenna. The radio pill technology was later used by Fields et al. [45] to study circulation time distributions (CTDs) in a pilot-scale air-lift bioreactor of approximately 0.5 m^3^, using a dilute xanthan solution as the model fluid. The CTD provides more information than the mean circulation time, or the mixing time, as the tail of the distribution may reveal long and critical circulation times. The CTD is valuable when examining the residence time in fluctuating microenvironments that the microorganisms may be exposed to, which is exactly what is necessary to understand the effect of heterogeneity [46]. The air-lift type bioreactors are similar to bubble column bioreactors but contain an inner cylinder which divides the flow into an upwards going fraction in the middle of the reactor, and a downwards going fraction at the side. The diameter of the applied radio pill measured only 10 mm. The CTD was determined by fixing an antenna at the middle height of the bioreactor. Thereby, each circulation was detected in the downwards flow stream at the side, where the radio pill came close to the antenna. The radio pill offered several advantages compared to existing technologies; it enabled flow analysis of a system which could otherwise not be visualized because of opaque media, bubbles or a stainless steel bioreactor wall, and it offered an alternative to traditional conductive tracers, which may alter the rheology of xanthan gum solutions [47].

The radio pill was later applied in a study of liquid circulation in an industrial bioreactor [26], [37], [38]. The examined system consisted of a 25 m^3^ bioreactor with a gas–liquid volume of 19 m^3^ which was agitated by two Rushton-type impellers. Antennas were placed inside the bioreactor in planar networks around the impellers to detect passages of the radio pill. Different circulation scenarios were thereby defined based on consecutive detections of the radio pills, from which the CTD and mean circulation time could be determined. In this study, the density of the radio pill was adjusted such that it was neutrally buoyant in water, while a gas–liquid dispersion was examined. Therefore, a significant part of the study was focused towards interpreting the data with the help of a model, which introduced several assumptions about the flow. Thereby, the effect from the “rate of fall”, which was determined experimentally in a glass cylinder, could be obtained from the gas fraction in the compartment and corrected from the CTD based on a “falling” probability.

During the late eighties and early nineties, positron cameras, electronics for timing and algorithms were developed in order to efficiently track and process data from positron-emitting particles [48], [49]. The technique is referred to as positron emission particle tracking (PEPT). Concurrently, a similar technology - computer-aided radioactive particle tracking (CARPT) - was developed and refined [50], [51]. The data obtained from these technologies are time series of 3D coordinates which reflect the particle trajectories. This Lagrangian information can be used to derive valuable flow characteristics, such as the CTD, or be converted into an Eulerian velocity map to examine the overall flow structure in the reactors [27], [28]. PEPT and CARPT have been used extensively in lab scale to better understand the flow in both bubble columns and stirred tank reactors [27], [28], [39], [51], [52]. The velocities and CTD obtained by CARPT have also been used to validate Lagrangian CFD predictions in a stirred tank reactor [28].

The tracer particles used in PEPT are as small as 100 µm [53]. The small size of the tracer particles makes them superior in terms of flow-following capabilities compared to the relatively large radio pill. Moreover, with PEPT and CARPT it is possible to obtain 3D trajectories, while the radio pill can only detect circulations. PEPT and CARPT are therefore much more suitable for detailed flow studies compared to the radio pills. However, they suffer from practical implications which limits the use in large-scale bioreactors. First, the detection of the particles requires that the examined system is encased by detectors. Second, both PEPT and CARPT rely on gamma radiation which suffers an intensity loss through stainless steel and liquid. It is therefore unlikely that the signal can cover the entire volume of a large bioreactor. Further drawbacks are the special precautions that may have to be taken when working with radiation [22].

### 3.1 Current state of sensor devices

#### 3.1.1 Sensor devices without position tracking

Sensor devices differ from the previously discussed Lagrangian measurement techniques in the sense that they can measure autonomously, which involves performing measurements in a controlled fashion, dictated by a microcontroller unit (MCU).

**Fig. 3:**
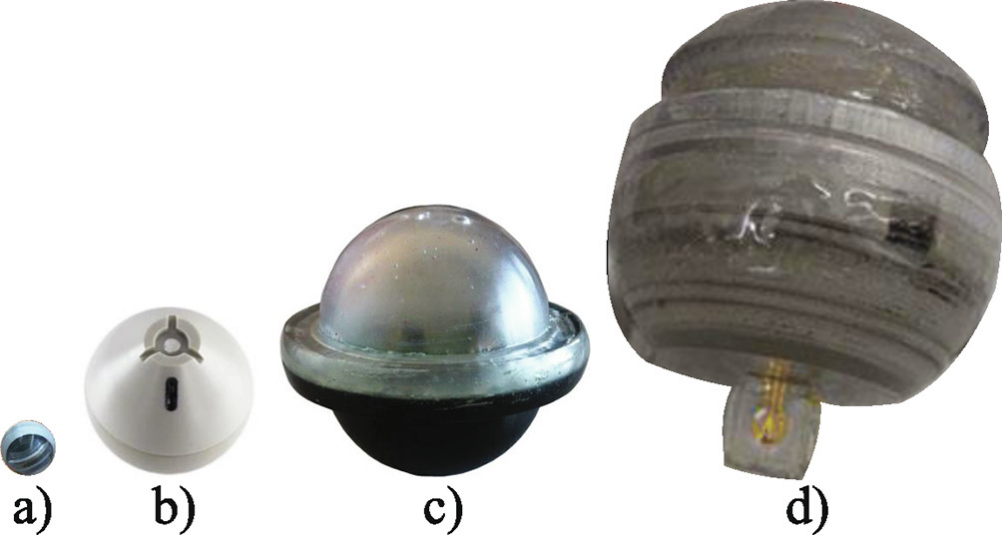
Instrumented sensor devices: a) Sens-o-sphere [58]. b) smartCAPS (pH version) [42]. c) Bio-capsule [43]. d) bPod [44].

Such sensor devices have been introduced in a commercial series of single parametric sensor devices named smartCAPS (Fig. 3b), which are capable of measuring either temperature, pH, acceleration, pressure or conductivity in bioprocess applications amongst others [42]. To the best of our knowledge, there is only literature available on early prototypes of these products. The earliest introduced prototypes, termed smart particles, were spheres with a diameter of 21 mm, which were able to measure temperature and wirelessly transmit the data via radio frequencies. The ‘smart particles’ were presented in a study which examined the convective flow in a rectangular container (40 × 40 × 10 cm) consisting of 25 mm thick poly(methyl methacrylate) with a cooled plate on the top and a heated plate on the bottom. Due to the fast response time of the temperature sensor of 0.06 s, it was possible to accurately describe the periodic temperature fluctuations measured by the sensor device. A successor to the smart particle, called smartPART, was built on the same concept as the smart particle, but included a three-dimensional (3D) accelerometer instead of a temperature sensor [54]. The research group presented a mathematical framework, which based on the acceleration signal enables the analysis of turbulent flow by identifying various flow structures, e.g. large vortex structures. This is demonstrated in a specialized mixing unit at lab-scale, which generates turbulent flow by two opposite counter rotating impellers [25]. The sensor device provides a simple approach to obtain insight on turbulent flows, as the characterization of a flow condition can be carried out in approximately 30 min and the sensor device can be used for 6 to 36 h depending on the power needed to transmit the acceleration signals. This makes the sensor device very versatile compared to other particle tracking methods, for instance PEPT, where the activity of the produced tracer decays over time and tracking time may be limited to 5 h [27]. However, the sensor device has a diameter of 25 mm, which in comparison to a PEPT particle in the micrometer scale, greatly limits the flow following capabilities and the length scale of the coherent turbulence structures which can be examined.

With respect to the application in a large-scale bioreactor, temperature gradients are typically not expected in processes maintained at constant temperature, as no significant variations have been measured at different positions in large-scale bioreactors [9], [55]. Therefore, a temperature sensor may not provide much new information. Information on turbulence structures, on the other hand, could provide valuable mixing insight as these structures are responsible for providing the energy to introduce new surface area, i.e. breakage of fluid elements and/or air bubbles. However, the application of the sensor devices is based on continuous data transmission which is not possible to maintain at any time in large-scale bioreactors, as the signal is dampened by the steel walls and the liquid volume.

Another sensor device, called the Sens-o-sphere, has been developed and is currently the smallest sensor device with its diameter of 7.9 mm (Fig. 3a) [56], [57]. The sensor device is capable of measuring temperature with a response time of 7 s and wirelessly transmit the data to a base station, using radio frequencies. The relatively long response time of the temperature sensor is due to the thermal conductivity of the sensor device shell, as the temperature sensor is not penetrating the shell. At the moment the research group developing the sensor device focuses on lab-scale experiments and validation of CFD models for different implementation scenarios [57], [58], together with the development of a localization system for the sensor devices based on inductive localization [59], which makes use of the wireless charging system in the sensor devices. Therefore, these sensor devices are not of immediate interest in large-scale applications, as temperature measurements are unlikely to reveal heterogeneity, as mentioned before. However, the sensor device may provide a non-invasive method to measure in applications where access to the process is limited, such as tubular reactors [56].

A sensor device capable of measuring conductivity, pH and concentrations of potassium, sodium and glucose, called the bio-capsule, has been presented by Todtenberg and co-workers [43]. The bio-capsule is a spherical sensor device with a diameter of 44 mm (Fig. 3c). One hemisphere is permeable to the fermentation broth and contains the sensors and the antenna used for data transmission while the other hemisphere is waterproof and contains the rest of the electronics required for operation of the sensor device. The device has been tested in a pilot-scale tubular glass photobioreactor containing a nutrient solution, where the focus was on the radio transmission. No details are presented with respect to sensor validation, except for the pH sensor, which was tested in reference solutions, showing voltage responses as expected [60]. The applied glucose sensor is an amperometric type sensor and due to the nature of the measurement method, reaction related mediator substances deposit on the electrode surface over time which attenuates the signal. Therefore, the estimated lifetime of the sensor device is limited to two weeks [60]. Based on the limited details presented on the sensor package in this study, it is difficult to assess the usefulness of this sensor device in large-scale bioreactors.

A recent study [44] aimed to develop a sensor device to study DO gradients in large-scale production of pharmaceuticals in stirred bioreactors. An electrochemical dissolved oxygen sensor was fabricated and integrated into a device with a diameter of 60 mm, named the bPod (Fig. 3d). The DO sensor was tested in a 2 L glass vessel where a steady state sensor response was achieved after 4 s. The sensor device was finally evaluated in a 10 L lab-scale bioreactor where DO measurements from the sensor device were compared with measurements collected by a commercial DO probe under different DO concentrations. The study showed promising results for DO measurements in sensor devices. However, this early prototype still suffered from a significant sensor drift and a short sensor lifetime.

Sensor devices have come a long way with respect to sensors which have been miniaturized and integrated, which is evident from the wide range of sensors in the presented sensor devices. However, little sensor validation and no lifeline studies are presented by the different research groups involved in such sensor development, which causes doubt about the robustness of the technologies. The sensor devices rely on wireless transmission, which suffers from some of the same limitations as PEPT and CARPT regarding signal loss in large stainless-steel bioreactors. However, the technologies seem promising as process analytical technologies for single use bioreactors in pharmaceutical manufacturing.

#### 3.1.2 Sensor devices with position tracking

The sensor devices presented in the previous section provide time series of given process variables. This information can be used to investigate the fluctuations in these process variables that microorganisms may encounter during their trajectories in the bioreactor, similar to information obtained from Euler-Lagrange simulations. However, no information about the trajectory itself is obtained which can complicate the data interpretation, especially regarding the flow following capabilities of the sensor devices, considering their relatively large size and possible inaccurate density. Hence, the sensor device may be spending most of its time in the bottom or the top of the liquid which cannot be inferred from the measurements.

**Fig. 4:**
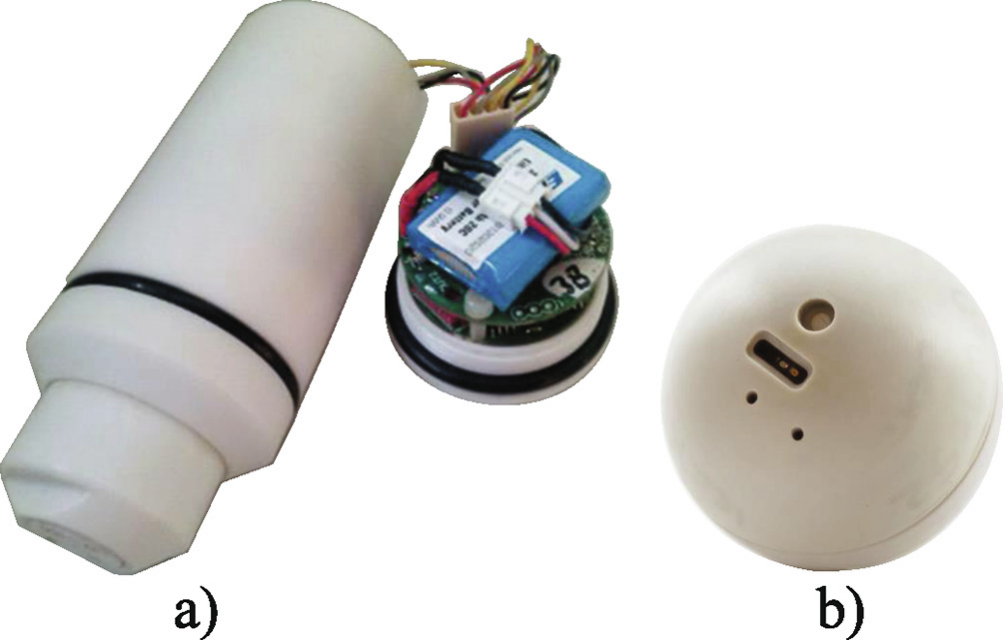
Sensor devices with axial position tracking: a) Sensor particle [61]. b) Fermsense 3D [66].

This problem has been solved by Thiele et al. [41] who have developed a sensor device for application in biogas digesters and wastewater treatment plants, which is capable of measuring temperature, pressure and 3D acceleration. In contrast to other developed sensor devices which are spherical, this sensor device has a cylindrical geometry (Fig. 4a). The advantage of this sensor device is the pressure measurement, which has been used to derive the immersion depth of the sensor device using Pascal’s principle*: Δh = ΔP/(ρg*), with *Δh* (m) being the immersion depth or the height of the fluid column above the measurement point, *ΔP* (Pa) is the hydrostatic pressure, *ρ* (kg/m^3^) is the fluid density and *g* (m/s^2^) is the gravitational acceleration [23]. The sensor device also differs from other sensor devices with respect to data collection strategy, which in this case focusses on storing the data internally rather than immediately transmitting it. Offline data collection may be the only option in large-scale bioreactors because wireless transmission through lossy transmission channels, such as metallic barriers, liquid conductivity, multiphase fluid and long distances, are currently infeasible. Since the original prototype, the sensor device has been further developed by the research group and has been equipped with a 3D magnetometer, 3D gyroscope and a buoyancy control unit [61]. The buoyancy control unit consists of an electronically controlled piston, which can rise to increase the volume of the sensor device and thereby reduce its density. With the buoyancy unit, the density can be adjusted automatically in stagnant process fluids, which therefore eliminates the need for disassembly and manual adjustment of the density. The buoyancy unit is also of importance with respect to retrieval of the sensor devices from a process. The sensor devices can be programmed to lower their density after a period of time, whereby each device floats to the liquid surface for retrieval. An offset in the density of 6% was found to force the sensor device to the liquid surface in fully turbulent flows [61]. This feature is very relevant for continuous fermentations run in an open and non-axenic way, while most axenic processes in industrial biotechnology applications are run as fed-batch processes. Here, the sensor device could potentially be collected by a filter in the outlet stream after the process has been ended.

Reinecke et al. from the same research group showed that axial residence profiles derived from the immersion depth of the sensor device can provide valuable information about the homogeneity in bioreactors. They showed that by changing the impeller clearance from 0.2 m to 0.324 m in a 1 m^3^ model fermenter with a highly viscous straw suspension, the homogeneity and axial dynamics in terms of axial velocity improved [23]. Similar results were shown in a pilot biogas digester of similar size, where better homogeneity could be obtained with roughly the same power input by increasing the impeller diameter and reducing the rotation speed [62].

The accelerometer was used to examine tendencies in the flow dynamics and to determine collisions of the sensor device with the impeller, which was detected by a peak in the acceleration measurements. It was demonstrated that the CTD could be determined from either the pressure measurements or the acceleration measurements [23]. In the case of acceleration, the frequency of the peaks was considered equal to the circulation time. The pressure approach utilized an arbitrary plane (of immersion depth). A circulation was then defined as consecutive intersections of the sensor device trajectory with this plane [23]. The approach to determine circulation time via measurements of hydrostatic pressure could in principle be used to determine the circulation rates between and inside the specific axial zones by modifying the location of the detection planes. Reinecke et. al compared and found good agreement between the average circulation times obtained by the sensor devices and the circulation times predicted by CFD simulations in a pilot-scale bioreactor. In this case, the sensor device was simulated as a dispersed phase using the Euler-Euler approach and the circulation time was derived from the streamlines [63]. The research group also proved that this concept could be applied to aerated bioreactors, by examining and comparing axial residence profiles and circulation times between a bubble column reactor and an air-lift reactor. A circulation time based analysis of the Peclet number, which describes the ratio of advective flow to diffusion, showed that a higher degree of back mixing was present in the bubble column reactor while the air-lift reactor was dominated by advective flow, which is expected from the enforced axial flow direction [62].

The magnetometer has been applied in the study of circulation times in a large 30 m^3^ pilot oval biogas digester. Measurements of the magnetic field enabled the sensor device to occasionally determine its position when it was in the proximity of a local position marker [24]. The research group is currently working on applying magnetic fields for data transmission and for expanding positioning with radial components [64], [65].

The use of sensor devices has also been validated in a real process, consisting of a 2077 m^3^ activated sludge basin at a wastewater treatment plant [62]. The sensor devices were successfully density adjusted and retrieved using the automated buoyancy control. The activated sludge basin was analyzed with respect to flow behavior in terms of axial velocity and inertial measurements, as well as thermal characteristics [62].

Another commercial sensor device is Fermsense 3D developed by Freesense ApS [66], [67]. This spherical sensor device with a diameter of 45 mm (Fig. 4b) is currently being commercially applied in a data-based mapping service of industrial fermentation processes [68]. FermSense 3D has recently been successfully deployed in an industrial scale bubble column, where temperature and pH were measured and linked to a pressure derived axial position over the duration of an entire *E. coli* fermentation [55]. The measurements revealed that no thermal gradients were present, but minor transient fluctuations were present in the pH in the lower part of the bioreactor due to the location of the ammonia gas addition [55].

The key features of the technologies reviewed in this section are summarized in Table 1.

**Table 1:** Overview of the sensor devices which have been developed for specific use in biotechnology applications.

Name	Diameter	Measured variables	Derived variables	References
Sens-o-sphere	7.9 mm	• Temperature	• Spatially resolved variables	[57], [69]
smartCAPS	25 mm	• Single variable quantified (temperature, acceleration, pH, conductivity or pressure)	• Spatially resolved variables • Hydrodynamic signature	[42]*
				[25], [70]
Bio-capsule	44 mm	• Potassium concentration, • Sodium concentration • Conductivity • pH • Glucose concentration	• Spatially resolved variables	[43], [60]
bPod	60 mm	• Dissolved oxygen	• Spatially resolved variables	[44]*
Sensor particle	52.8 mm**	• Temperature • Pressure • Acceleration • Magnetic field	• Spatially resolved variables • Circulation time • Axial position • Axial velocity	[41], [61], [71]
FermSense 3D	45 mm	• Temperature • pH • Acceleration • Pressure	• Spatially resolved variables • Flow patterns	[55], [66]*

* Not from peer reviewed literature.

** Volume equivalent particle diameter (cylinder).

**Fig. 5:**
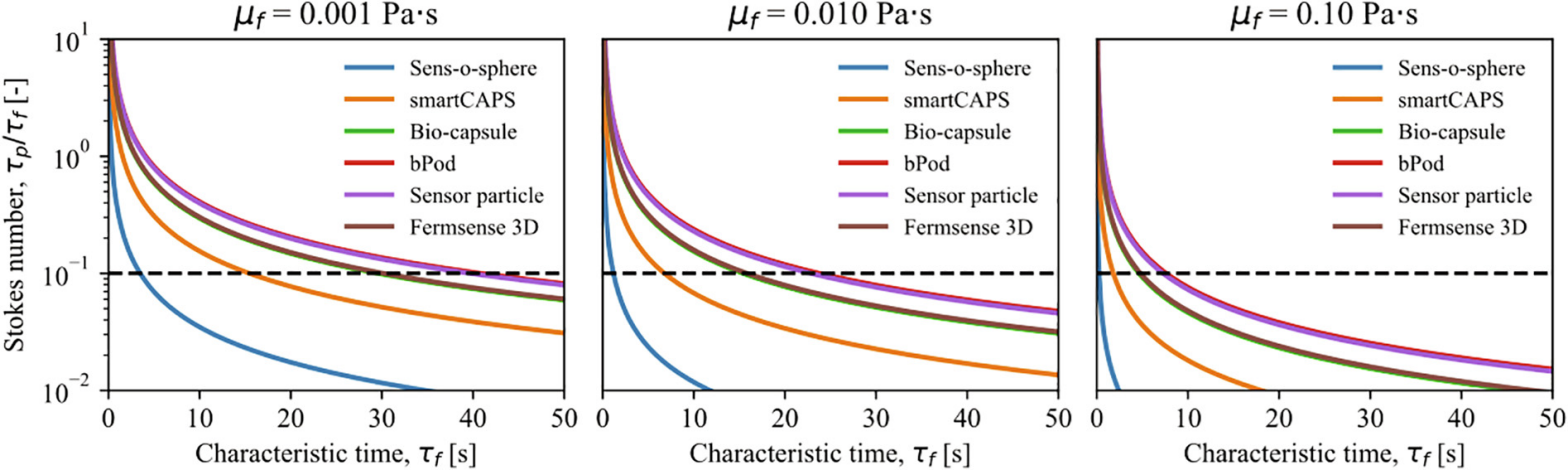
Stokes number as a function of the characteristic time for the reviewed sensor devices. The relationship is presented for water (0.001 Pa s) and for a low (0.01 Pa s) and a high viscosity (0.1 Pa s) fermentation. The figure serves as a comparison of how the diameter of the sensor devices and the fluid viscosity affects the flow following capabilities. The presented values are only rough estimates as the velocity of the sensor devices compared to the fluid is assumed to be constant, at 0.05 m/s. In addition, perfect buoyancy (ρ_p_/ρ_f_) = 1 is assumed.

The Stokes numbers for the reviewed sensor devices as a function of the characteristic time for flow in water and two common fermentation viscosities are shown in Fig. 5 [16].

The Stokes number is calculated from Eqs. (1), (2), with the drag coefficients estimated by correlations for spherical and non-spherical particles by Levenspiel and Haider [72]. The estimations of the Stokes number in Fig. 5 only exemplify the effects of diameter differences and should not be taken as accurate predictions, as a constant particle to fluid velocity difference of 0.05 m/s is assumed, together with perfect buoyancy (ρ_p_/ρ_f_) = 1.

The time scale, i.e. the characteristic time, of the phenomena that the sensor devices can accurately represent are drastically reduced with reductions in the sensor device diameter and increases in the viscosity. Because of the drag coefficient, the effect of increased diameter is greater than the proportional relationship of particle diameter and relaxation time shown in Eq. (3). Due to the similarities in the particle diameters, the Bio-capsule and Fermsense 3D show identical behaviors and it is difficult to distinguish the lines in the figure. The same applies to the bPod sensor device and the sensor particle. Some examples of characteristic times which may be of interest for sensor devices in bioreactors are the impeller diameter to tip speed ratio, which implies an intermediate mixing scale between turbulence and bulk mixing, or the circulation time in the bioreactor, which implies bulk mixing of the process [23].

It should be mentioned that most viscous fermentation broths exhibit shear thinning behavior, which means that the viscosity varies spatially depending on the local shear rates in the bioreactor [16]. Therefore, the flow following capabilities may also vary locally.

Sensor devices with position tracking have proven useful in the study of flow and mixing. However, the information that can be obtained about position is limited to the axial direction together with a few local reference points, e.g. the impeller or locally installed position markers. Nevertheless, the practical simplicity and the scalable positioning methods make them promising technologies for large-scale bioreactors.

## 4 Discussion

The overview of sensor devices in Table 1 shows that a range of variables can currently be measured with sensor devices. Temperature sensors are present in most of the sensor devices, even though temperature gradients are supposedly not a big issue in large-scale bioreactors. However, it should not be ruled out that very viscous broths or bioreactors with poorly designed cooling systems may suffer from temperature gradients. The main reason that temperature sensors are so widespread is probably because the underlying technology is simple and inexpensive in terms of space and battery usage. Temperature sensors are usually also accompanying the majority of sensors by default, as temperature compensation is required for most sensor technologies. Several of the developed sensor devices also include a pH sensor. Measurements of the pH value are interesting in relation to heterogeneity because accumulation of acidic by-products during cell growth demands pH control, usually by addition of base [73]. Therefore, in the case of deficient mixing in the bioreactor, considerable transient fluctuations of pH are expected near the base addition zone, which have already been demonstrated using sensor devices [55]. Spatial gradients in pH have been shown to promote the formation of by-products and to reduce the viability of the production organism [73], [74], [75]. Glucose concentration and DO concentration are arguably the most important variables in relation to gradients, as both may be rapidly consumed at high cell growth rates and are continuously added to the fermenter in most industrial fermentations. Consequently, their spatial distribution depends on the mixing and mass transfer conditions in the bioreactor. Despite their great importance, glucose and DO sensors are uncommon in sensor devices. The reason is that there are considerable challenges associated with the technologies. One of these challenges is the response time of the sensors. In a bioreactor with axial velocities up to two meters per second, as determined in a CFD study of a large-scale aerated bioreactor [17], a sensor response time of half a second is required to get a spatial resolution of one meter in the axial direction. The reported response time for the DO sensor was approximately 4 s [44]. No response time was reported for the glucose sensor, but is expected to be even longer than 4 s based on response times of currently available technologies [76]. Response times much slower than a couple of seconds render the sensors unsuitable for measuring differences between different zones in high velocity processes, for example in industrial fermentation processes, as they will merely measure an averaged contribution of the variable for the entire bioreactor. However, sensors with slower response times are still relevant for processes with lower fluid velocities, which also have a higher probability of experiencing gradients. Another challenge is that the most widely utilized glucose sensors are enzyme-based (glucose oxidase) [77] and therefore cannot undergo steam sterilization, as the high temperatures will inactivate the enzymes immobilized in the sensor. Steam sterilization is the most widespread approach to ensure an axenic environment in industrial fermentation processes, and therefore the introduction of the sensor device after the bioreactor has been sterilized poses a potential contamination risk to the process.

The sensor device from Thiele et al. and the Fermsense 3D sensor device offer the possibility to link the measured variables to an axial position. Even though the flows in the bioreactors are highly three-dimensional, the axial position by itself is valuable, as a significant part of the dynamics in large-scale bioreactors are axial, because the height to diameter ratio of such bioreactors is usually greater than two [10], [15], [16], [17], [18], [19]. The axial position has also proven valuable for characterizing axial flow and to determine CTDs in the bioreactors. The drawback of using the hydrostatic pressure to calculate the immersion depth is that knowledge about the fluid density is required. The bubbles themselves do not affect the pressure measurements, as the total pressure inside the bubbles is equal to the pressure of the surrounding fluid. While the broth density is easily determined from extracted samples of the broth, the apparent density of gas–liquid dispersions is more difficult to estimate accurately in bioreactors [30]. This can therefore result in inaccuracies of the calculated immersion depth by Pascal’s principle. For example, in a situation where the apparent density of the fluid is estimated to 800 kg/m^3^, but the actual apparent density is 900 kg/m^3^. From Pascal’s principle it follows that for a given measured pressure difference, *Δh_actual_ = Δh_estimate_*(*ρ_estimate_*/*ρ_actual_*). Therefore, the estimated height of the fluid above the sensor device, i.e. the immersion depth of the sensor device, is 1.125 times greater than the actual height of the fluid above the sensor device. Correspondingly, the velocities obtained from the derivative are inaccurate by a factor of 1.125. This also means that the total error will be largest at the largest pressure difference, i.e. when the sensor device is furthest away from the reference pressure at the liquid surface.

In addition, the apparent density may vary locally, e.g. over the liquid height of the bioreactors. This could especially be the case in tall bubble column bioreactors. Here, the gas expansion and changing equilibrium between bubble coalescence/break-up with hydrostatic pressure, affects the superficial gas velocity and the gas hold-up. The actual extent of occurrence of gas hold-up gradients in large bioreactors is uncertain, but CFD simulations show variations of the gas hold-up from 18% to 24 % in the majority of the volume in a 125 m^3^ bubble column reactor, while a greater difference exists when including zones in the vicinity of the top and bottom [19]. This means that a measured pressure difference of 7500 Pa may correspond to a change in axial position of approximately one meter for the lower part of the reactor where the gas hold-up is 24%, while the same pressure difference corresponds to a change in the axial position of 0.93 m at the upper part of the reactor where the gas hold-up is 18%.

Accurate axial flow characteristics and CTDs can be directly compared to, and serve as validation, for CFD simulations with particle tracking. The current sensor devices are still limited in terms of important variables that can be measured, such as concentrations of substrates, products of by-products. CFD models which may have been validated by the axial flow profiles, CTDs or even DO profiles obtained from sensor devices could be used to model some of these variables that are challenging to measure. The technologies can therefore synergize to provide a better understanding about gradients in general and their impact on the production organisms. To the best of our knowledge, no studies that compare sensor device measurements with CFD simulations have been published thus far, besides the comparison of circulation times obtained by sensor devices and CFD simulations by Reinecke et al. [62]. In the future, more studies concerning CFD modelling of large-scale bioreactors could greatly benefit from comparisons with sensor device measurements.

An extension of the axial position with the two radial components (i.e. 3D positioning) is required to also be able to examine gradients in the radial direction. This is relevant because the addition of substrate, acid/base or even oxygen may be located somewhere away from the center axis. The three-dimensional trajectories are also the ideal input for validation of CFD models. However, a 3D positioning system has not yet been realized for large-scale bioreactors as the stainless-steel tanks and the turbulent opaque broths with a large fraction of dispersed gas prevent visibility and seriously limit transmission options. Inertial navigation systems (INS) based on measurements of acceleration and angular velocities in three dimensions using MEMS sensors have been studied in detail for applications such as pedestrian navigation and unmanned drone technology [78], [79], [80]. This type of positioning system is interesting because the position can in theory be derived from on-board sensors. The position and absolute rotation from an initial state can be obtained through double integration of acceleration and integration of angular velocity, respectively. However, in practice the MEMS sensors are subject to non-linear noise, which if integrated, quickly accumulates to large errors in the position, making the estimates valid for short periods of time only. Buntkiel et al. have proposed to use an INS in bioreactors with the sensor particle (Table 1) with additional inputs from a magnetometer and the pressure sensor to the positioning system [81]. The system has shown to produce accurate results in an experimental setup, but is yet to be investigated in situ (i.e. for sensor devices circulating in bioreactors) [64], [81]. A concept which combines the INS with radio frequency time-of-flight measurements has also been proposed for the use in large-scale biogas reactors. The position can be determined using radio signals when the sensor devices are at the liquid surface, while the INS takes over once the sensor devices are submerged. When the sensor devices rise to the surface once again, the position determined by the INS can be checked against the time-of-flight measurements [82].

It should be mentioned that measurements of a variable in time without information about the position could still be relevant for the validation of lifeline studies with Euler-Lagrange simulations, i.e. with a statistical comparison of the trajectory measurements. However, to the best of our knowledge, such studies have also not been published at the time of writing. It should also be emphasized that the sensor devices presented in Table 1 are 3–4 orders of magnitude larger than the cells of commonly used production organisms. It is therefore expected that the sensor device’s representation of the cell trajectories is limited with respect to accuracy. A microorganism with a diameter of 1 μm has a Stokes number that is approximately eight orders of magnitudes lower than a spherical sensor device with a diameter of 45 mm, when the viscosity is equal to that of water. This means that the microorganisms will practically behave as the liquid in the bioreactors. While this is not true for sensor devices, they are generally able to follow the bulk circulation flows. It can be seen from Fig. 5 that under high viscosity conditions, flow tracing accuracy errors of less than 1% can be obtained for circulation times >10 s. This is the case even for the largest sensor devices. Viscous processes with high characteristic times because of low velocity fields or large volumes are therefore ideal systems for macroscopic sensor devices. Specific types of bioreactors, such as biogas digesters, fit this profile well with even higher viscosities than exemplified in Fig. 5. Reinecke et al. demonstrated that the relatively large sensor device (sensor particle, Table 1), should be able to follow the circulation flows with an error that is less than 1%, even in a model system at pilot scale [23]. An acceptable tracing accuracy (St < 1) may still be obtained for most circulation flows with circulation times >5 s by the large sensor devices in Table 1, in the worst case of water-like viscosity. Still, there should be continued motivation towards reducing the size of the sensor devices to improve accuracy for a wide range of processes, especially when interpreting the measurements from a cell lifeline perspective. Statistical analysis of measured variables and the determined circulation times could provide indications of the time distribution of microorganisms in critical zones. However, the results should be interpreted with the flow following capabilities of the sensor devices in mind. For example, the residence time in zones of high energy input may potentially be biased towards lower residence times because the flow structures in these zones are much smaller and will entrain the microorganisms, but not the sensor devices.

Fermentation processes with changing density profiles could be dealt with by deploying multiple sensor devices with a range of densities and by interpreting several datasets corresponding to different periods of the process. In addition to affecting the Stokes number, the discrepancy between sensor device density and the fluid density also affects buoyancy of the sensor devices. As presented previously, Barneveld et al. measured falling velocities of the radio pill to correct the measured circulation times in a gas–liquid dispersion [26]. A gas holdup of 20% resulted in a falling velocity of the radio pill of 0.27 m/s, when the radio pill was adjusted to be neutrally buoyant in water. As with the Stokes number, falling/rising velocity calculations may only serve as an initial estimate, because the drag force is not directly opposing the gravity when the fluid is in motion [83]. In practice, major discrepancies between the sensor device density and fluid density are expected to be readily visible from the axial residence times of the sensor devices. As an example, Reinecke et al. purposefully forced the devices to the liquid surface by introducing a density offset of 6% [61]. This was confirmed by measurements of hydrostatic pressure, which showed that the sensor devices spent most of the time near the liquid surface. To be able to reach the surface, the rising velocity should exceed the dynamics in the velocity field of the flow.

To validate the flow following capabilities of the sensor devices rather than relying on estimations of the Stokes number, it would be obvious to compare the derived trajectories and velocities of the sensor devices with established methods, such as PEPT and CARPT, which utilize particles with diameters in the micrometer range. To the best of our knowledge, such studies have not yet been published. Cross-checking with PEPT and CARPT might not be an easy task as the macroscopic sensor devices may not be compatible with the same systems as PEPT or CAPRT, and vice versa. PEPT and CARPT provide accurate flow predictions, but the technologies are not readily scalable to large bioreactors, while sensor devices will suffer from inaccurate flow predictions in smaller bioreactors due to poor flow following capabilities and effects from boundaries, e.g. collisions with impellers, walls, baffles etc. A pilot scale bioreactor with low velocity field and high viscosity could prove to be a compatible system for both technologies.

## 5 Summary and outlook

Computational fluid dynamics (CFD) with coupled microbial kinetics or advanced cell models allow for predictions about gradients and their consequences in large-scale bioreactors. However, it is often not possible to demonstrate their validity with experimental data, because the access to high quality data in large-scale bioreactors is very limited. During production, only measurements from fixed sensors or samples extracted from sample ports are available, so complex custom-built sensor setups are sometimes developed to get a better understanding of the environment in the bioreactors. Flow following sensor devices offer a completely different perspective, which enables the acquisition of detailed spatial information in a relatively simple way. Direct measurement of variables linked to a position enables us to quantify potential occurrence of gradients and together with derived parameters, such as circulation time, this can provide the necessary input to validate the CFD models.

The sensor devices can improve the knowledge of large-scale bioreactors by:

• Confirming the presence and the spatial distribution of gradients
• Determine flow characteristics of the macroscopic flow, such as circulation time and velocity field.
• Examine the evolution of process parameters in space and time, which can be interpreted as what a microorganism experiences (i.e. lifeline analysis).

With respect to confirmation of spatial gradients, there are still some limitations related to the diversity of the variables that can be spatially resolved. Sensor devices with DO and glucose sensors are emerging, and the focus here should be on improving the technologies with a faster response time and increased precision and/or higher accuracy.

The determination of flow characteristics would benefit from the development of smaller sensor devices, leading to better flow following capabilities. Future studies that validate sensor device trajectories against established methods, such as PEPT and CARPT, would provide a better understanding of the limitations of sensor devices with different shapes and sizes. Currently, the spatial information that can be obtained is limited to the axial direction and the development of a more complete positioning system will enable characterization of the bioreactor environment in even greater detail, which can significantly improve our knowledge on large-scale bioreactors. The microorganism lifeline analysis will benefit greatly from both sensor development and improvement in the flow following capabilities.

It is obvious that the analyses from macroscopic sensor devices are not flawless, but compared to the currently available methods, they are a major step towards a better understanding of the environment in large-scale bioreactors. The sensor devices should therefore also be applied in validating CFD models, which are currently considered a state-of-the-art tool for understanding large-scale bioreactors.

Furthermore, a large quantity of a new type of data is being generated by the sensor devices. Therefore, novel data-processing methods are needed to fully exploit the available data from different sensor outputs and convert it into interpretable figures and visualizations.

It is also worth mentioning that many of the presented sensor devices are considered prototypes, and some general features should be developed further for the application in large-scale bioreactors, such as mechanical resistance, heat resistance, chemical resistance and sampling capacity to meet the requirements of the production facilities.

Many of these issues can, and will be resolved in the future, but it should be clear that these developments may require a considerable amount of time, before a flexible and versatile technology is available that can provide spatially resolved measurements of a range of relevant parameters in large-scale reactors. The sensor device technology will continue to benefit from technological advances within MEMS, microsensors and battery technology.

## CRediT authorship contribution statement

**Jonas Bisgaard:** Writing - original draft, Conceptualization, Visualization. **Monica Muldbak:** Resources, Writing - review & editing. **Sjef Cornelissen:** Writing - review & editing. **Tannaz Tajsoleiman:** Writing - review & editing. **Jakob K. Huusom:** Supervision, Writing - review & editing. **Tue Rasmussen:** Supervision, Writing - review & editing. **Krist V. Gernaey:** Supervision, Writing - review & editing.

## Declaration of Competing Interest

Tue Rasmussen, Tannaz Tajsoleiman (industrial Postdoc), and Jonas Bisgaard (industrial PhD student) are full-time employees at Freesense, a company that has a commercial interest in sensor devices. Monica Muldbak, Jakob K. Huusom, and Krist V. Gernaey are employed at the Technical University of Denmark, with no commercial interests in the sensor devices. Sjef Cornelissen is employed at Novozymes and has an interest in the sensor devices as an end user of the technology.

## References

[1] Deloitte. Opportunities for the fermentation-based chemical industry. An analysis of the market potential and competitiveness of North-West Europe. 2014.

[2] Crater J., Lievense J.C. (2018). Scale-up of industrial microbial processes. FEMS Microbiol Lett.

[3] Lübbert A., Beroviĉ M., Nienow A.W. (2005). Bubble columns and airlift loop bioreactors. Biochem. Eng. Princ., Ljubljana, Birmingham: Faculty of Chemistry and Chemical Technology.

[4] Enfors SO. Continuous and fed-batch fermentation. In: Beroviĉ M, Nienow AW, editors. Biochem. Eng. Princ., 2005, p. 146–70.

[5] Stocks S.M. (2013). Industrial enzyme production for the food and beverage industries: process scale up and scale down. Microb Prod Food Ingredients, Enzym Nutraceuticals, Elsevier Ltd..

[6] Enfors S.-O., Jahic M., Rozkov A., Xu B., Hecker M., Jürgen B. (2001). Physiological responses to mixing in large scale bioreactors. J Biotechnol.

[7] George S., Larsson G., Olsson K., Enfors S.-O. (1998). Comparison of the Baker's yeast process performance in laboratory and production scale. Bioprocess Eng.

[8] Oosterhuis N.M.G., Kossen N.W.F. (1984). Dissolved oxygen concentration profiles in a production-scale bioreactor. Biotechnol Bioeng.

[9] Manfredini R., Cavallera V., Marini L., Donati G. (1983). Mixing and oxygen transfer in conventional stirred fermentors. Biotechnol Bioeng.

[10] Larsson G., Törnkvist M., Wernersson E.S., Trägårdh C., Noorman H., Enfors S.-O. (1996). Substrate gradients in bioreactors: origin and consequences. Bioprocess Eng.

[11] Larsson G., Törnkvist M. (1996). Rapid sampling, cell inactivation and evaluation of low extracellular glucose concentrations during fed-batch cultivation. J Biotechnol.

[12] Pigou M., Morchain J., Fede P., Penet M.-I., Laronze G. (2017). An assessment of methods of moments for the simulation of population dynamics in large-scale bioreactors. Chem Eng Sci.

[13] Haringa C., Mudde R.F., Noorman H.J. (2018). From industrial fermentor to CFD-guided downscaling: what have we learned?. Biochem Eng J.

[14] Noorman H. (2011). An industrial perspective on bioreactor scale-down: what we can learn from combined large-scale bioprocess and model fluid studies. Biotechnol J.

[15] Haringa C., Tang W., Deshmukh A.T., Xia J., Reuss M., Heijnen J.J. (2016). Euler-Lagrange computational fluid dynamics for (bio)reactor scale down: an analysis of organism lifelines. Eng Life Sci.

[16] Bach C. (2019). Modelling of Gradients in Large Scale Bioreactors.

[17] Morchain J., Gabelle J.-C., Cockx A. (2014). A coupled population balance model and CFD approach for the simulation of mixing issues in lab-scale and industrial bioreactors. AIChE J.

[18] Kuschel M., Siebler F., Takors R. (2017). Lagrangian trajectories to predict the formation of population heterogeneity in large-scale bioreactors. Bioengineering.

[19] Siebler F., Lapin A., Hermann M., Takors R. (2019). The impact of CO gradients on C. ljungdahlii in a 125 m3 bubble column: mass transfer, circulation time and lifeline analysis. Chem Eng Sci.

[20] Lapin A., Müller D., Reuss M. (2004). Dynamic behavior of microbial populations in stirred bioreactors simulated with Euler-Lagrange methods: traveling along the lifelines of single cells. Ind Eng Chem Res.

[21] Tinka A., Rafiee M., Bayen A.M. (2013). Floating sensor networks for river studies. IEEE Syst J.

[22] Groen D.J. (1994). Macromixing in Bioreactors.

[23] Reinecke S., Deutschmann A., Jobst K., Kryk H., Friedrich E., Hampel U. (2012). Flow following sensor particles—validation and macro-mixing analysis in a stirred fermentation vessel with a highly viscous substrate. Biochem Eng J.

[24] Reinecke S.F., Jobst K., Hampel U. (2017). Untersuchung der Hydrodynamik von ovalen Biogasreaktoren mit instrumentierten Strömungsfolgern. Chemie Ing Tech.

[25] Zimmermann R., Fiabane L., Gasteuil Y., Volk R., Pinton J.-F. (2013). Characterizing flows with an instrumented particle measuring Lagrangian accelerations. New J Phys.

[26] van Barneveld J., Smit W., Oosterhuis N.M.G., Pragt H.J. (1987). Measuring the liquid circulation time in a large gas—liquid contactor by means of a Radio Pill. 1. Flow pattern and mean circulation time. Ind Eng Chem Res.

[27] Chiti F., Bakalis S., Bujalski W., Barigou M., Eaglesham A., Nienow A.W. (2011). Using positron emission particle tracking (PEPT) to study the turbulent flow in a baffled vessel agitated by a Rushton turbine: improving data treatment and validation. Chem Eng Res Des.

[28] Khopkar A.R., Rammohan A.R., Ranade V.V., Dudukovic M.P. (2005). Gas–liquid flow generated by a Rushton turbine in stirred vessel: CARPT/CT measurements and CFD simulations. Chem Eng Sci.

[29] Mavros P. (2001). Flow visualization in stirred vessels. Chem Eng Res Des.

[30] Hofmeester J.J.M. (1988). Gas hold-up measurements in bioreactors. Trends Biotechnol.

[31] Middleton J.C. (1979). Measurement of circulation within large mixing vessels. Resour Energy.

[32] Tropea C., Yarin A.L., Foss J.F. (2007). Particle-based techniques. Handb. Exp. Fluid Mech..

[33] Crowe C.T., Schwarzkopf J.D., Sommerfeld M., Tsuji Y. (2012). Properties of dispersed phase flows. Multiph. Flows with Droplets Part.

[34] Turton R., Levenspiel O. (1986). A short note on the drag correlation for spheres. Powder Technol.

[35] Bhattacharyya B. Introduction. Electrochem. Micromach. Nanofabrication, MEMS Nanotechnol., William Andrew; 2015, p. 1–23. DOI:10.1016/b978-0-323-32737-4.00001-3.

[36] Mann R., Mavros P.P., Middleton J.C. (1981). A structured stochastic flow model for interpreting flow-follower data from a stirred vessel. Trans Inst Chem Eng.

[37] Oosterhuis NMG. Scale-up of bioreactors, a scale-down approach. Ph.D. dissertation. TU Delft, 1984.

[38] van Barneveld J., Smit W., Oosterhuis N.M.G., Pragt H.J. (1987). Measuring the liquid circulation time in a large gas—liquid contactor by means of a radio pill. 2. Circulation time distribution. Ind Eng Chem Res.

[39] Fangary Y.S., Seville J.P.K., Barigou M. (1999). Flow studies in stirred tanks by positron emission particle tracking (PEPT). Inst Chem Eng.

[40] Wadke P.M., Hounslow M.J., Salman A.D. (2005). The ‘Smart’ Sphere. Chem Eng Res Des.

[41] Thiele S., Schöne S., Voigt F., Da Silva M.J., Hampel U. (2009). Design of a neutrally buoyant self-powered multi-parameter sensor for data logging in flow applications. Proc IEEE Sens.

[42] SmartINST. In Situ Wireless Measurements 2015. https://webma9021.wixsite.com/smartinstnew/smartcaps-in-situ-wireless-measurem (accessed January 31, 2020).

[43] Todtenberg N, Klatt J, Schmitz-Hertzberg ST, Jorde F, Schmalz K. Wireless sensor capsule for bioreactors. 2013 IEEE MTT-S Int. Microw. Work. Ser. RF Wirel. Technol. Biomed. Healthc. Appl. IMWS-BIO 2013 - Proc., IEEE Computer Society; 2013. DOI:10.1109/IMWS-BIO.2013.6756236.

[44] Stine J. BPOD: A wireless integrated sensor platform for continous localized bioprocess monitoring. Master’s thesis. University of Maryland, 2019. DOI:10.13016/UDQB-PWBV.

[45] Fields P.R., Mitchell F.R.G., Slater N.K.H. (1984). Studies of mixing in a concentric tube air-lift reactor containing xanthan gum by means of an improved flow follower. Chem Eng Commun.

[46] Amanullah A., Buckland B.C., Nienow A.W. (2004). Mixing in the fermentation and cell culture industries. Handb. Ind. Mix..

[47] Charles M. (1978). Technical aspects of the rheological properties of microbial cultures. Adv. Biochem. Eng., Springer.

[48] Bemrose C.R., Fowles P., Hawkesworth M.R., O'Dwyer M.A. (1988). Application of positron emission tomography to particulate flow measurement in chemical engineering processes. Nucl Instrum Methods Phys Res, Sect A.

[49] Parker D.J., Broadbent C.J., Fowles P., Hawkesworth M.R., McNeil P. (1993). Positron emission particle tracking - a technique for studying flow within engineering equipment. Nucl Instrum Methods Phys Res, Sect A.

[50] Lin J.S., Chen M.M., Chao B.T. (1985). A novel radioactive particle tracking facility for measurement of solids motion in gas fluidized beds. AIChE J.

[51] Devanathan N., Moslemian D., Dudukovic M.P. (1990). Flow mapping in bubble columns. Chem Eng Sci.

[52] Yang Y.B., Devanathan N., Duduković M.P. (1993). Liquid backmixing in bubble columns via computer-automated radioactive particle tracking (CARPT). Exp Fluids.

[53] Bridgwater J., Forrest S., Parker D.J. (2004). PEPT for agglomeration?. Powder Technol.

[54] Zimmermann R, Fiabane L, Gasteuil Y, Volk R, Pinton J-F (2013). Measuring Lagrangian accelerations using an instrumented particle. Phys Scr.

[55] Zahn J, Bisgaard J, Rasmussen T, Kulhanek A, Halter M. Scale-up and Optimization of a Fermentation Process for Production of Propanediol in a Bubble Column Bioreactor 2019. https://sim.confex.com/sim/raft13/meetingapp.cgi/Paper/40673 (accessed February 3, 2020).

[56] Lüke T., Büker M.J., Hedayat C., Lenk F., Lauterbach T., Gernandt T. (2017). Sens-o-Spheres | A concept for location independent acquisition of process measurement signals. Int. Conf. Exhib. Integr. Issues Miniaturized Syst..

[57] Lauterbach T., Lüke T., Büker M.J., Hedayat C., Gernandt T., Moll R. (2019). Measurements on the fly– Introducing mobile micro-sensors for biotechnological applications. Sens Actuators, A.

[58] Lauterbach T., Ziebart N., Bley T., Walther T., Lenk F. (2019). Mobile Sensoren für die Biotechnologie – Ortsunabhängige, miniaturisierte Prozessmessung. Chem Ing Tech.

[59] Lange S. Reliable localisation in a bioreactor. Microelectron News 2019:3. https://www.mikroelektronik.fraunhofer.de/en/Presse/NachrichtenUebersicht/MikroelektronikNachrichten/ENAS_Zuverlaessige_Ortung_im_Bioreaktor.html (accessed February 3, 2020).

[60] Todtenberg N., Schmitz-Hertzberg S.T., Schmalz K., Klatt J., Jorde F., Jüttner B. (2015). Autonomous sensor capsule for usage in bioreactors. IEEE Sensors J.

[61] Reinecke S.F., Hampel U. (2016). Instrumented flow-following sensor particles with magnetic position detection and buoyancy control. J Sens Sens Syst.

[62] Reinecke S.F., Hampel U. (2017). Investigation of bioreactors by instrumented flow-following sensor particles. AMA Conf.

[63] Reinecke S.F., Deutschmann A., Jobst K., Hampel U. (2017). Macro-mixing characterisation of a stirred model fermenter of non-Newtonian liquid by flow following sensor particles and ERT. Chem Eng Res Des.

[64] Buntkiel L, Reinecke S. Inertiale Lage- und Bewegungsverfolgung für instrumentierte Strömungsfolger zur Strömungscharakterisierung. 14 Dresdner Sensor-Symposium 2019:52–8. DOI:10.5162/14dss2019/3.5.

[65] Hott M., Hoeher P.A., Reinecke S.F. (2019). Magnetic communication using high-sensitivity magnetic field detectors. Sensors (Switzerland).

[66] Freesense ApS. Our technology 2020. http://freesense.dk/ (accessed February 1, 2020).

[67] Andersen M. Kloge golfbolde til bioindustrien. Dynamo 2018:04–7.

[68] Freesense ApS. Fermsense 3D. Data-based tank mapping service for the Bioprocess industry 2020. https://www.freesense.dk/data-based-fermentation-mapping (accessed February 3, 2020).

[69] Lüke T, Büker MJ, Hedayat C, Lenk F, Lauterbach T, Gernandt T, et al. Sens-o-Spheres | A concept for location independent acquisition of process measurement signals. In Int. Conf. Exhib. Integr. Issues Miniaturized Syst. 2017, SSI 2017, Mesago Messe Frankfurt GmbH; 2017, p. 67–74.

[70] Shew W.L., Gasteuil Y., Gibert M., Metz P., Pinton J.F. (2007). Instrumented tracer for Lagrangian measurements in Rayleigh-B́nard convection. Rev Sci Instrum.

[71] Thiele S., Da Silva M.J., Hampel U. (2010). Autonomous sensor particle for parameter tracking in large vessels. Meas. Sci. Technol..

[72] Haider A., Levenspiel O. (1989). Drag coefficient and terminal velocity of spherical and nonspherical particles. Powder Technol.

[73] Lara A.R., Galindo E., Ramírez O.T., Palomares L.A. (2006). Living with heterogeneities in bioreactors. Mol Biotechnol.

[74] Amanullah A., McFarlane C.M., Emery A.N., Nienow A.W. (2001). Scale-down model to simulate spatial pH variations in large-scale bioreactors. Biotechnol Bioeng.

[75] Onyeaka H., Nienow A.W., Hewitt C.J. (2003). Further studies related to the scale-up of high cell densityescherichia coli fed-batch fermentations: the additional effect of a changing microenvironment when using aqueous ammonia to control pH. Biotechnol Bioeng.

[76] Rahman M.M., Ahammad A.J.S., Jin J.H., Ahn S.J., Lee J.J. (2010). A comprehensive review of glucose biosensors based on nanostructured metal-oxides. Sensors.

[77] Chen C., Xie Q., Yang D., Xiao H., Fu Y., Tan Y. (2013). Recent advances in electrochemical glucose biosensors: a review. RSC Adv.

[78] Nilsson J.O., Gupta A.K., Handel P. (2014). Foot-mounted inertial navigation made easy. Int Conf Indoor Position Indoor Navig.

[79] Nilsson J.O., Skog I., Händel P., Hari K.V.S. (2012). Foot-mounted INS for everybody - an open-source embedded implementation. Rec - IEEE PLANS, Position Locat Navig Symp.

[80] Chambers A., Scherer S., Yoder L., Jain S., Nuske S., Singh S. (2014). Robust multi-sensor fusion for micro aerial vehicle navigation in GPS-degraded/denied environments. Proc Am Control Conf.

[81] Buntkiel L., Reinecke S., Hampel U. (2020). Towards 3D-motion tracking of instrumented flow followers in large vessels. SMSI Conf. – Sens. Meas. Sci. Int..

[82] Annas S., Elfering M., Jantzen H.-A., Scholz J., Janoske U., Heller A. (2014). 14. Rostocker Bioenergieforum. Rühr- und Mischvorgänge Biogasanlagen Potentiale und Erfolgschancen, Universität Rostock.

[83] Chhrabra R., Shankar V. (2017). Motion of particles in a fluid. Coulson Richardson’s. Chem Eng.

